# Forecasting Under-5 Mortality Rate in Somalia to 2030: a comparative analysis of univariate and multivariate ARIMAX models

**DOI:** 10.3389/fpubh.2026.1783096

**Published:** 2026-04-08

**Authors:** Suhaib Mohamed Kahie Seiman, Mustafe Mohamoud Abdi, Abdisalam Hassan Muse, Khadar Mowlid Abdi, Khadar Jama Dahir, Omran Salah, Saralees Nadarajah

**Affiliations:** 1 School of Postgraduate Studies and Research, Amoud University, Borama, Somalia; 2 Department of Freshman, Amoud University, Borama, Somalia; 3 Faculty of Education, Amoud University, Borama, Somalia; 4 Research and Innovation Centre, Amoud University, Borama, Somalia; 5 Department of Mathematics, University of Manchester, Manchester, United Kingdom

**Keywords:** ARIMA-TBATS hybrid models, ARIMAX, conflict intensity, SDG 3.2, Somalia, TBATS, Under-5 Mortality Rate (U5MR)

## Abstract

**Background:**

Accurate forecasting of the Under-5 Mortality Rate (U5MR) is essential for monitoring health progress and informing policy interventions in conflict-affected regions. In Somalia, achieving the Sustainable Development Goal (SDG) 3.2 target of reducing child mortality to at least 25 deaths per 1,000 live births by 2030 remains a formidable challenge due to persistent socio-political instability and economic shocks.

**Methods:**

Utilizing a two-phase analytical approach, the study first evaluated 11 time-series models (6 single and 5 hybrid) based on historical U5MR data from 1960 to 2023. In the second phase, a multivariate ARIMAX framework (1989–2023) was implemented to incorporate exogenous drivers, including total conflict-related fatalities, GDP per capita, and immunization coverage. Model performance was validated using MAPE, sMAPE, and Theil’s *U* statistics, supplemented by rigorous diagnostic tests such as Shapiro–Wilk and Breusch–Pagan.

**Results:**

The univariate analysis identified the TBATS and hybrid ARIMA–TBATS models as the superior forecasting tools, with the latter selected for its robustness in handling Somalia’s high data volatility. The multivariate ARIMAX model revealed that conflict fatalities are a highly significant predictor of mortality ( p<0.001 ), while GDP per capita serves as a significant negative determinant ( p=0.0128 ). The ARIMAX model projects a U5MR of 86.29 deaths per 1,000 live births by 2030, while trend-based univariate models project a plateau at 109.78. Both projections indicate that Somalia remains significantly off-track to meet the SDG 3.2 target.

**Conclusion:**

The findings underscore a concerning stagnation in child mortality reduction. Achieving international benchmarks in Somalia requires a dual focus on intensifying health interventions and ensuring national security stability. These data-driven insights offer critical evidence for policymakers to design resilient strategies for child survival in the region.

## Introduction

Under-five mortality (U5M), defined as the probability of a child dying between birth and exactly 5 years of age, remains a critical indicator of a nation’s health, social development, and economic stability. It reflects the nutritional status of children, the knowledge of mothers regarding health, the level of immunization, and the availability of maternal and child health services (1, 2). Reducing this rate is a central tenet of the global development agenda, specifically Sustainable Development Goal (SDG) 3.2, which targets ending preventable deaths of newborns and children under 5 years of age, with all countries aiming to reduce under-5 mortality to at least 25 per 1,000 live births by 2030 (3, 4). Despite global progress, current trends suggest that without intensified action, over 48 million children under five are projected to die between 2020 and 2030, half of them newborns (5). Achieving the SDG target would avert nearly 11 million of these deaths, yet significant barriers such as poverty, lack of education, and weak health systems continue to impede progress (5, 6).

Historically, regions outside of Africa have witnessed substantial declines in child mortality driven by economic growth and improved healthcare infrastructure. In East Asia and the Pacific, and parts of South Asia, mortality rates have plummeted due to targeted interventions against infectious diseases. For instance, China achieved a 37.1% decline in U5MR between 2009 and 2015, shifting the epidemiological profile where leading causes of death transitioned from infectious diseases like pneumonia to neonatal causes such as preterm birth complications and congenital abnormalities (7). Similarly, in Bangladesh, deaths due to infectious causes have declined significantly, though drowning remains a persistent cause of death for children aged 1–4 years (8). However, disparities remain; while wealthier nations have minimized preventable deaths, lower-middle-income countries in Asia and Latin America still face challenges with neonatal mortality and disparities between rural and urban populations (9–12).

Conversely, the African continent, particularly Sub-Saharan Africa (SSA), continues to bear the highest burden of child mortality globally. In 2019, 1 in 13 children in SSA died before their fifth birthday, a rate 15 times higher than in high-income countries (2, 13). While the region has seen reductions since the Millennium Development Goals (MDGs) era, the pace has been insufficient to meet SDG targets (5). Nigeria, for example, ranked among the top nations with high under-five mortality, recording 117 deaths per 1,000 live births in 2019, driven by malaria, pneumonia, and diarrhea (14, 15). The region faces unique challenges including the “unfinished agenda” of infectious diseases combined with emerging issues like climate change impacts and conflict, which exacerbate malnutrition and weaken health systems (16–18).

In East Africa, the trajectory of child survival (0–4 years) has been volatile, influenced heavily by environmental shocks and socio-political instability. Countries like Ethiopia, Kenya, and Tanzania have made commendable strides through community health extension programs, yet mortality rates remain high compared to global standards (19, 20). Research indicates that in this region, neonatal deaths occupy a higher proportion of under-five deaths, often linked to sepsis, birth asphyxia, and prematurity (21, 22). Furthermore, recurrent droughts in the Horn of Africa have led to severe malnutrition crises, directly stalling progress in reducing child mortality rates (23, 24). The interdependence of maternal health and child survival is pronounced here, where maternal education and access to antenatal care are critical determinants of survival (25, 26).

Somalia presents one of the most challenging contexts for child survival in the world. Decades of civil conflict, absence of a central functioning government for long periods, and recurrent famine have resulted in some of the highest child mortality rates globally. Historical data reveals extreme volatility; sharp spikes in mortality were observed around 1991–1992 during the civil war and again in 2011 during the famine (27, 28). Unlike other regions where trends follow a smoother decline, Somalia’s data is characterized by “shocks” that disrupt health service delivery and immunization programs (29). While recent years have shown a gradual downward trend, the rate remains critically high, necessitating robust forecasting to understand future trajectories amidst ongoing instability and climate risks (30–32).

The persistence of high under-five mortality is driven by a complex interplay of socioeconomic, biological, and environmental factors. Systematic reviews highlight that maternal education, the mother’s age at childbirth, birth interval, and household wealth status are significant covariates of child survival (1, 33). Specifically, children born to mothers with no formal education or those residing in rural areas with poor sanitation are at exponentially higher risk (34, 35). Furthermore, limited access to interventions such as skilled birth attendance, vaccination, and clean cooking fuels exacerbates the risk of death from preventable causes like pneumonia and diarrhea (36, 37). In conflict-affected settings like Somalia, these structural factors are compounded by the destruction of health infrastructure, making accurate prediction and resource allocation vital for survival (38, 39).

### Study objectives

1. To identify the most robust time-series model (single vs. hybrid) for projecting Somalia’s U5MR.
2. To investigate the impact of socio-political (conflict), Measles Immunization and economic (GDP) drivers on child survival using an ARIMAX framework.
3. To evaluate Somalia’s progress toward the SDG 3.2 target (25 deaths per 1,000 live births) by 2030.

## Materials and methods

The study employed a time-series research design, which falls under the umbrella of longitudinal research designs. Time-series analysis involves evaluating a substantial sequence of observations collected on the same variable in consecutive order over a specified period. This study utilizes historical data on the Under-5 Mortality Rate (U5MR) in Somalia, spanning from 1960 to 2023. Unlike previous absolute count measures, U5MR is defined as the probability of a child dying before reaching age five, expressed per 1,000 live births.

The data were primarily obtained from the Our World in Data database, which integrates estimates from the United Nations Inter-Agency Group for Child Mortality Estimation (UN IGME). To facilitate the multivariate ARIMAX analysis, supplementary exogenous data including conflict-related fatalities from the Uppsala Conflict Data Program (UCDP) and GDP per capita and immunization coverage from the World Bank were also integrated into the dataset. The final dataset comprises annual U5MR values for each year within the 64-year study period, providing a robust foundation for both univariate and multivariate forecasting.

### Variable selection and rationale

The selection of variables for this study was strategically designed to capture the complex interplay between demographic trends, socio-economic factors, and institutional shocks that define child survival in Somalia. The primary dependent variable is the Under-5 Mortality Rate (U5MR), defined as the probability of a child dying before reaching age five, expressed per 1,000 live births. This rate-based metric was prioritized over absolute death counts to ensure alignment with the Sustainable Development Goal (SDG) 3.2 targets and to provide a standardized measure that accounts for population growth dynamics. To investigate the external drivers of U5MR volatility, three exogenous variables were integrated as regressors in the multivariate ARIMAX framework. First, Total Conflict-related Fatalities (sourced from the Uppsala Conflict Data Program) was selected as a proxy for conflict intensity, reflecting the impact of civil instability and security shocks on health service delivery. Second, GDP per capita (current US$) was utilized to represent national economic capacity and household wealth status, which are critical determinants of access to nutrition and medical care. Third, Measles Immunization Coverage (% of children ages 12–23 months) served as an indicator of healthcare infrastructure performance and the efficacy of preventative health interventions. Although environmental factors such as Annual Precipitation (mm) were initially considered due to their historical association with famine-related mortality, they were excluded from the final quantitative models as the available historical time-series data exhibited zero annual variance (constant values), rendering them unsuitable for statistical regression. Consequently, the influence of environmental shocks is addressed qualitatively in the study’s discussion and formally acknowledged as a data limitation.

### Stationarity testing with ADF, PP, and KPSS tests

In time series research, it is essential to determine whether a given time series is stationary. To assess this, the Augmented Dickey–Fuller (ADF) test, the Phillips–Perron (PP) test, and the Kwiatkowski–Phillips–Schmidt–Shin (KPSS) test were applied.

### Time series model development

The study employs a range of univariate time series modeling techniques to forecast child deaths (ages 0–4) in Somalia. These techniques include:

### ARIMA model

To model the linear temporal dynamics of under-5 mortality in Somalia, we employed the Autoregressive Integrated Moving Average (ARIMA) framework, originally proposed by Box and Jenkins (40). This method is particularly effective for univariate data that exhibits non-stationarity, as it transforms the series through differencing (*d*) before applying autoregressive (*p*) and moving average (*q*) parameters. The general mathematical representation of the ARIMA (*p*, *d*, *q*) process applied to the mortality variable $y_{t}\;$ is given by:


$$\begin{array}{l} (1-\varphi_{1}L-\varphi_{2}L^{2}-\ldots -\varphi_{p}L^{p}){\left(1-L\right)}^{d}\\ y_{t}=(1+\theta_{1}L+\theta_{2}L^{2}+\ldots +\theta_{q}L^{q})\varepsilon_{t} \end{array}$$ (1)

Where $y_{t}$ represents the time series of child deaths (ages 0–4), L is the lag operator, and $\varphi_{i}$ and $\theta_{i}$ are the autoregressive and moving average coefficients, respectively. The parameter p denotes the order of the autoregressive component, d is the degree of differencing applied to make the series stationary, and q is the order of the moving average component. $\varepsilon_{t}$ is a white noise error term.

Model implementation was executed using the `auto.arima()` function within the forecast package in R. This algorithm streamlines the selection of model parameters (*p*, *d*, *q*) by employing the Hyndman–Khandakar stepwise search method (41–43). To ensure parsimony and prevent overfitting, the Akaike Information Criterion (AIC) was utilized as the primary selection metric, where the model yielding the lowest AIC score was deemed the optimal fit for the under-5 mortality series. Following model estimation, we conducted rigorous diagnostic checks to validate assumptions; this included analyzing the standardized residuals and autocorrelation function (ACF) plots, alongside the Ljung-Box test to confirm that the residuals resembled white noise.

### ARFIMA

To test for and model long-range dependence within the time series, we extended the analysis using the ARFIMA process (44). This method generalizes the standard ARIMA framework by permitting a non-integer (fractional) differencing parameter, denoted as d. This fractional integration is crucial for identifying if the mortality rates follow a long-memory process, where the correlation structure is stronger and lasts longer than what is captured by standard autoregressive models. The general equation is defined as follows:


$$\begin{array}{l} (1-\varphi_{1}L-\varphi_{2}L^{2}-\ldots -\varphi_{p}L^{p}){\left(1-L\right)}^{d}\\ (1-\theta_{1}L-\theta_{2}L^{2}-\ldots -\theta_{q}L^{q})y_{t}=\varepsilon_{t}, \end{array}$$ (2)

Where $y_{t}$ represents the time series of child deaths (ages 0–4), L is the lag operator, $\varphi_{i}$ and $\theta_{i}$ are the autoregressive and moving average coefficients, p is the order of the autoregressive component, d is the degree of fractional differencing, and q is the order of the moving average component.

### Theta model

We also incorporated the Theta method, which gained prominence for its superior performance in the M3 Forecasting Competition (45). This technique decomposes the seasonally adjusted mortality series into two components known as “theta lines” which modify the local curvature of the data. The first line removes the curvature to capture the long-term trend, while the second amplifies it. Hyndman et al. (46) later demonstrated that the standard Theta method is statistically equivalent to Simple Exponential Smoothing (SES) with a drift term. This makes it an ideal benchmark for capturing the gradual downward or upward trends in child mortality. The forecasting equation is given by:


$$y_{t+1}=(1+\theta )y_{t}+\mu +\varepsilon_{t+1}$$ (3)

Where $\left(y_{t}\right)$ is the timeseries, (θ ) is the parameter representing the slope, (μ) is the drift term, and ( $\varepsilon_{t+1}$ ) is the error term.

### ETS model

To accommodate both linear and non-linear patterns in the mortality trajectory, we utilized the Exponential Smoothing (ETS) state-space framework (46). This approach is distinct from traditional smoothing methods because it explicitly models the underlying process using three stochastic components: Error (E), Trend (T), and Seasonality (S). The algorithm automatically evaluates a taxonomy of up to 30 possible model configurations by permuting these components as either Additive (A), Multiplicative (M), or None (N). For the Somali child mortality dataset, this flexibility is crucial for handling potential heteroscedasticity (changing variance) or dampened trends over time. The general state-space equations governing the observation and state transition are defined as:


$$y_{t}=w(x_{t-1})+r\;(x_{t-1})\varepsilon_{t}$$ (4)

And


$$x_{t}=f(x_{t-1})+g\;(x_{t-1})\varepsilon_{t}$$ (5)

In these equations, the coefficients w , f , and g represent the respective parameters, while $\varepsilon_{t}$ denotes the Gaussian white noise series. Equation 4 describes the relationship between the lagged state variable $x_{t-1}$ and the observed variable $y_{t}$ , while Equation 5 represents the transitional equation describing the evolution of states over time. To implement the ETS model, the `ets()` function from the “forecast” package was utilized (47).

### NNAR model

While models like ARIMA assume linear relationships, epidemiological data often contain complex, non-linear interactions due to external shocks. To capture these dynamics in the Somali under-5 mortality dataset, we implemented the Neural Network Autoregression (NNAR) model. Specifically, we utilized a feed-forward multilayer perceptron (MLP) architecture with a single hidden layer. In this framework, the lagged values of the time series serve as inputs analogous to autoregressive terms but are processed through non-linear activation functions (sigmoid) in the hidden nodes before generating the output. The mathematical formulation of the network, as described by Yusof and Kane (48), relates the output $Y_{t}$ to the inputs via the following equation:


$$Y_{t}=\omega_{0}+\sum_{j=1}^{Q}\omega_{g}g\;(\;\omega_{\mathrm{oj}}+\sum_{i=1\;}^{p}\omega_{i,j}\;y_{t-1}\;)+e_{t}$$ (6)

In this expression $,y_{t}$ corresponds to the forecasted mortality value, while the vector ( $y_{t-1},$ $\ldots ,y_{t-p})$ constitutes the input layer. The terms $\omega_{\left\{i,j\right\}}\;$ and $\omega_{j}\;$ represent the synaptic weights connecting the input, hidden, and output layers, respectively. The structural complexity is defined by p input nodes and Q neurons in the hidden layer.

To implement this empirically, we utilized the `nnetar()` algorithm within the R forecast environment [Cite Hyndman]. This function automates the architectural design for seasonal data, denoted as $\text{NNAR}{\left(p,P,k\right)}_{m}.$ Here, p and P refer to the non-seasonal and seasonal lag orders, while m indicates the frequency. The network topology was optimized heuristically: the non-seasonal lag p was selected based on the optimal linear autoregressive model, while the number of hidden nodes was determined by the rule $k=\frac{p+P+1}{2}$ . This approach balances model capacity with generalization, preventing the network from memorizing noise.

### TBATS model

To address potential non-linearities and complex seasonal behaviors that standard ARIMA or ETS models might miss, we employed the TBATS framework (49). The acronym stands for Trigonometric seasonality, Box-Cox transformation, ARMA errors, Trend, and Seasonality. This method is particularly advantageous for epidemiological time series exhibiting non-constant variance (heteroscedasticity). It achieves this by automatically selecting a Box-Cox transformation parameter ( ω ) to stabilize the variance before modeling the seasonality using Fourier series. This allows for a flexible representation of cyclic patterns without requiring integer seasonality constraints. The measurement equation governing this process is defined as:


$$Y_{t}^{\omega }=l_{t-1}+\varphi \;b_{t-1}+\sum_{t=1}^{T}s_{t-m_{i}}^{i}+d_{t}$$ (7)

In this equation, $y_{t}^{\omega }$ represents the observation after applying the Box-Cox transformation with parameter ω , while $y_{t}$ is the original observation at time t. $l_{t}$ lt denotes the local level, φ represents the damped trend, b represents the long-run trend, T indicates the seasonal pattern, $s_{t}^{i}$ denotes the i−th seasonal component, $m_{i}$ represents the seasonal period, and $d_{t}$ corresponds to the ARMA(*p*, *q*) process for the residuals. The TBATS model was identified using the tbats () function included in the “forecast” package.

### Hybrid models

In addition to the individual time series models, the study explores hybrid modeling approaches that combine multiple techniques to improve forecasting accuracy. A comprehensive set of 5 hybrid time-series models was developed by systematically combining the single techniques: ARIMA, ETS, TBATS, Theta, and NNAR. These models ranged from simple two a-model combinations (e.g., ARIMA-ETS, ARIMA-TBATS) to complex multi-model ensembles (ARIMA-ETS-Theta-NNAR-TBATS). These models were created using the `hybrid Model ()` function available in the “forecast Hybrid” package in R (50). The hybrids were constructed using equal weights to integrate the strengths of different models, aiming to capture both linear and non-linear trends and seasonality in the child mortality (ages 0–4) data. The detailed process of model development using these functions has been previously described (46, 51–55).

In the construction of the hybrid ensembles, an equal weighting strategy was employed to integrate the constituent time-series models. This decision was informed by several methodological considerations. First, in highly volatile and conflict-affected contexts like Somalia where data are subject to extreme stochastic shocks such as civil unrest and recurring famines equal weighting serves as a robust hedge against the risk of overfitting, a common drawback of performance-based weighting schemes. Second, empirical literature on the ‘forecast combination puzzle’ suggests that simple averages often outperform more complex weighting methods in environments characterized by high uncertainty and structural instability. By utilizing equal weights, this study ensures that the hybrid models remain resilient to historical anomalies while maintaining a balanced synthesis of both linear and non-linear dynamics from the underlying individual models.

### Multivariate ARIMAX modeling

To address the limitations of univariate time-series models and incorporate the impact of external shocks such as conflict intensity and socio-economic fluctuations this study implemented a Multivariate ARIMAX (Autoregressive Integrated Moving Average with Explanatory Variables) framework. Unlike standard ARIMA models that rely solely on historical patterns of the dependent variable, the ARIMAX model integrates exogenous covariates ( $X_{t}$ ) to enhance forecasting precision and explanatory power (43, 56). This approach is particularly effective for modeling mortality rates in conflict-affected contexts like Somalia, where abrupt shifts in child survival are often driven by identifiable external factors rather than inherent trends alone (57). The ARIMAX model can be mathematically expressed as follows:


$$y_{t}=\mu +\sum_{i=1\;}^{k}\beta_{i}x_{i,t}+\mathrm{\eta t}\;$$

In this model, $y_{t}$ represents the Under-5 Mortality Rate (U5MR) at time t , while $\beta_{i}$ denotes the coefficients of the exogenous variables ( $x_{i,t}$ ), which in this study specifically include Total Conflict-related Fatalities, GDP per capita, and Measles Immunization Coverage. Furthermore, $\eta_{t}$ serves as the ARIMA error term tasked with capturing the underlying stochastic process; this term is subsequently decomposed into its autoregressive (*p*), integrated (*d*), and moving average (*q*) components, following the classical time-series framework established by Box and Jenkins (40).

The inclusion of these exogenous variables is supported by epidemiological literature. For instance, conflict fatalities serve as a critical proxy for security shocks that disrupt healthcare delivery and maternal-child health services (38). GDP per capita represents the economic determinants of child survival, such as household nutritional capacity and access to medical resources (1). Furthermore, immunization coverage is a vital indicator of public health infrastructure performance (24). The model was implemented in the R statistical environment using the auto.arima() function with the xreg argument, ensuring that the optimal orders of (*p*, *d*, *q*) were selected based on the lowest Akaike Information Criterion (AIC) (43). This dual-stage modeling approach (univariate trend analysis followed by multivariate causal analysis) provides a comprehensive assessment of Somalia’s progress toward SDG 3.2.

### Model evaluation

To assess the performance of the time series models, the dataset is divided into training and validation sets. The models are fitted to the training data, and their forecasting accuracy is evaluated by comparing the predicted values against the actual child death (ages 0–4) values in the validation set. Performance metrics specifically the Mean Absolute Percentage Error (MAPE), Symmetric Mean Absolute Percentage Error (sMAPE), and Theil’s *U* statistic are calculated to quantify the accuracy of the models.

#### Selection of best-fitting models

Based on the model evaluation results, the best-fitting time series models are identified. Models with lower MAPE, sMAPE, and Theil’s *U* values are considered superior in terms of forecasting accuracy. The selected models provide the basis for generating future forecasts of child deaths (ages 0–4) in Somalia.

#### Analysis of trends and seasonality

The time series models and their forecasts are analyzed to identify trends and patterns in child mortality (ages 0–4) over the historical period. This analysis provides insights into the dynamics of child survival and helps understand the long-term trends and potential factors influencing them, such as instability or environmental shocks.

#### Implications for policy and intervention

The findings from the time series analysis and forecasting serve as a basis for formulating policy recommendations and interventions. The historical volatility and projected trends in child deaths (ages 0–4) highlight the urgency for implementing control strategies and effective measures, particularly regarding healthcare access, nutrition programs, and famine prevention, both within Somalia and in vulnerable communities.

In summary, this study employs a range of univariate time series models, including ARIMA, ETS, Theta, NNAR, ARFIMA, and TBATS, as well as hybrid modeling approaches, to forecast child deaths (ages 0–4) in Somalia. The models are evaluated and compared using performance metrics, and trends in child mortality (ages 0–4) are analyzed. The results provide valuable insights for policymakers and stakeholders to develop effective strategies and interventions for reducing child mortality (ages 0–4) and promoting public health outcomes in Somalia.

### Model adequacy and diagnostic tests

To ensure the internal validity and statistical reliability of the developed models, rigorous diagnostic checks were performed on the residuals. The Shapiro–Wilk test (58) was utilized to assess the normality of residuals, while the Breusch-Pagan test (59) was conducted to check for heteroscedasticity. Additionally, the Durbin-Watson test (60) and the Ljung-Box *Q*-statistic (61) were employed to examine residual autocorrelation. For the multivariate analysis, the Variance Inflation Factor (VIF) was calculated to ensure the absence of multicollinearity among the exogenous predictors (62), thereby validating the model’s robustness and ensuring that the coefficient estimates were not biased by inter-variable correlations.

### Forecast and model performance evaluation

To assess the predictive performance and reliability of the models developed in this study, three robust evaluation metrics were employed: Mean Absolute Percentage Error (MAPE), Symmetric Mean Absolute Percentage Error (sMAPE), and Theil’s *U* statistics (53). MAPE was selected for its high interpretability in reporting forecast accuracy, while sMAPE was utilized to provide a symmetric measure of percentage error, thereby mitigating potential bias in instances of lower mortality values. Additionally, Theil’s *U* statistic was included to evaluate forecast quality relative to a naïve benchmark, where a value less than one indicates that the model outperforms a simple no-change forecast (54). The evaluation process was conducted in two distinct phases: first, univariate models were validated using the long-term Under-5 Mortality Rate (U5MR) dataset spanning 1960–2023, partitioned into a training set (1960–2013) and a testing set (2014–2023). Second, the multivariate ARIMAX framework was evaluated for the period 1989–2023 to specifically capture the explanatory and predictive power of exogenous drivers such as conflict intensity and economic fluctuations. The selection of the optimal forecasting tool was based on the minimization of these error metrics across both phases (55). Finally, the superior models were utilized to project Somalia’s U5MR trajectory for 2024–2030, facilitating a direct comparison with the Sustainable Development Goal (SDG) 3.2 target of 25 deaths per 1,000 live births.

### Software and research process

**Figure 1:**
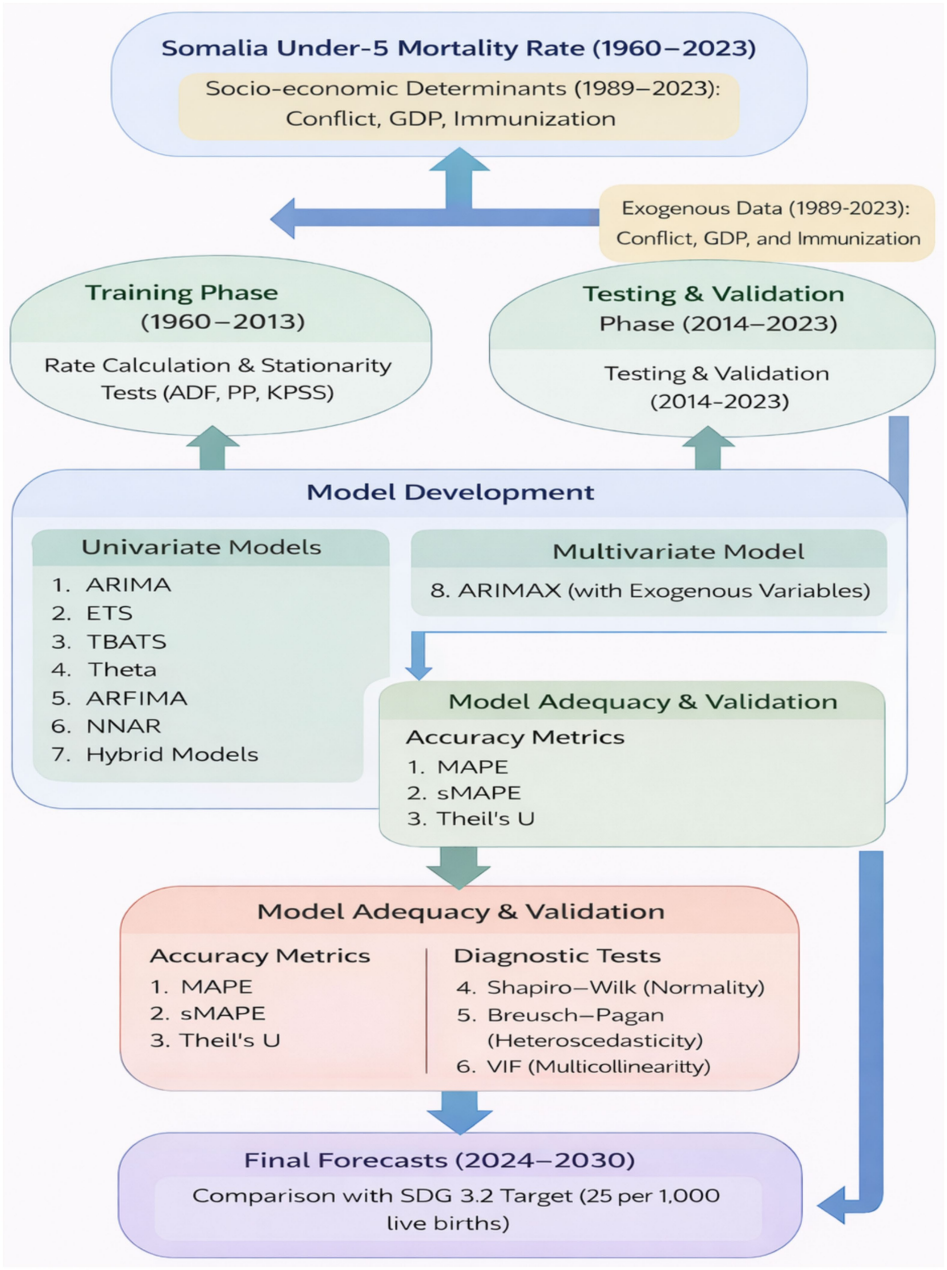
Methodological framework of the two-phase forecasting approach, integrating univariate trend analysis (1960–2023) and multivariate ARIMAX modeling (1989–2023). The procedure includes rigorous model validation using accuracy metrics (MAPE, sMAPE, Theil’s *U*) and diagnostic testing (Shapiro–Wilk, Breusch-Pagan, VIF) to evaluate Somalia’s trajectory toward the SDG 3.2 target.

The R statistical software and various R packages including “psych,” “tseries,” “TSstudio,” “forecast,” “Metrics,” “plotly,” “forecast Hybrid,” and “nnfor” were utilized for data analysis, time series modeling, and forecasting. The process of analyzing, modeling, and forecasting time series data consisted of several steps. These steps encompassed: (i) examining and transforming the data into a time series format, (ii) assessing the stationarity of the data utilizing ADF, PP, and KPSS tests and applying differencing techniques to achieve stationarity, (iii) identifying the final model through a model development process, (iv) evaluating the performance of the developed model, and (v) generating forecasts for child deaths (ages 0–4) based on the final model. The complete dataset containing the number of child deaths (ages 0–4) was divided into separate training and testing datasets. Prediction models such as ARIMA, ETS, TBATS, Theta, ARFIMA, NNAR, and hybrid models were constructed using the training data. To assess the performance of these models, error measures specifically Mean Absolute Percentage Error (MAPE), Symmetric Mean Absolute Percentage Error (sMAPE), and Theil’s *U* statistic were calculated and utilized for comparing the accuracy of the prediction models. Figure 1 illustrates these sequential steps.

## Results

### Data description

#### Descriptive statistics of the data

The descriptive statistics provide a comprehensive summary of the Under-5 Mortality Rate (U5MR) in Somalia for the period 1960–2023. The variable represents the annual number of deaths of children under 5 years of age per 1,000 live births. The dataset consists of 64 annual observations. As shown in the analysis, the mean U5MR over the study period is 194.41, with a standard deviation of 61.88, indicating significant temporal variability in child survival. The median value stands at 185.65, while the trimmed mean is 187.68, which helps account for extreme fluctuations in the series. The mortality rates span a wide range, from a minimum of 103.44 to a maximum of 453.52 deaths per 1,000 live births. The distribution of the data is characterized by a strong positive skewness (2.33) and a leptokurtic distribution with a high kurtosis value of 7.26. These metrics signify the presence of “heavy tails” and extreme outliers, directly reflecting catastrophic historical shocks most notably the sharp mortality spike in 1991–1992 during the onset of the Somali civil war. The standard error of the sample mean is approximately 7.73. Overall, these statistics highlight a high-risk environment with extreme volatility, underscoring the challenges Somalia faces in achieving stable declines in child mortality and aligning with international targets like SDG 3.2 (see Table 1).

**Table 1:** Descriptive statistics of the Under-5 Mortality Rate (U5MR) per 1,000 live births in Somalia (1960–2023).

Variable	# of Obs. (*n*)	Mean	SD	Median	Trimmed mean	MAD
Data	64	194.41	61.88	185.65	187.68	39.02

Variable	Minimum	Maximum	Range	Skewness	Kurtosis	SE
Data	103.44	453.52	350.08	2.33	7.26	7.73

#### Visual representation of the data

**Figure 2:**
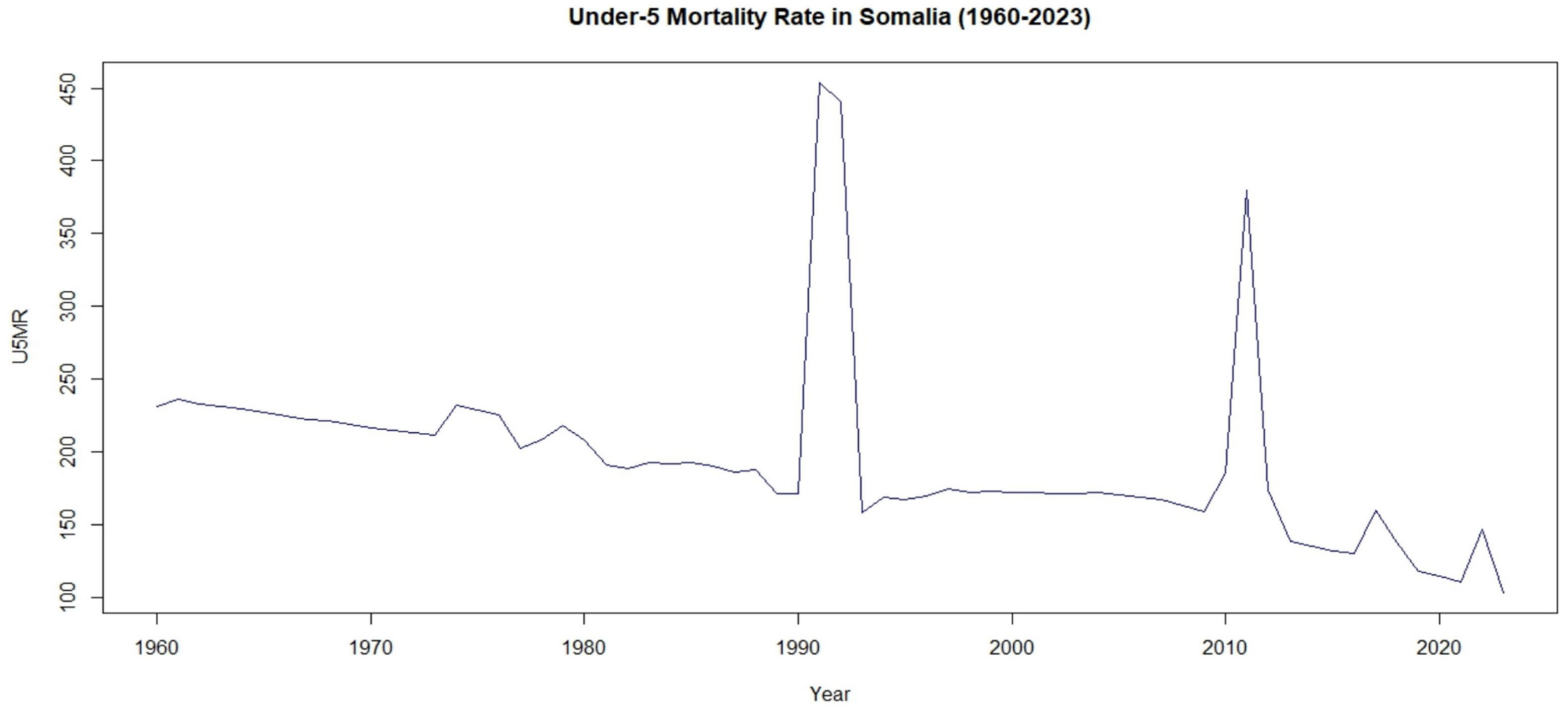
Time-series plot of the Under-5 Mortality Rate (U5MR) in Somalia (1960–2023).

Figure 2 illustrates the temporal trajectory of the Under-5 Mortality Rate (U5MR) in Somalia from 1960 to 2023. The trend depicted in Figure 2 indicates a general downward trajectory in child mortality over the 64-year study period, falling from approximately 231 deaths per 1,000 live births in 1960 to 103.4 by 2023. However, this decline has been far from consistent.

The U5MR series is characterized by extreme volatility and significant stochastic shocks. Most notably, a catastrophic spike is observed during 1991–1992, where mortality rates peaked at 453.5 per 1,000, coinciding with the peak of the Somali civil war and the subsequent collapse of the healthcare system. A second prominent peak occurred in 2011, reaching 379.5 per 1,000, which corresponds to the severe famine and ongoing insecurity in the region. These fluctuations underscore the profound vulnerability of child survival in Somalia to socio-political instability and environmental crises, highlighting that progress toward the SDG 3.2 target remains fragile and subject to external shocks.

#### Data stationarity tests

In time-series analysis, ensuring stationarity is a prerequisite for reliable modeling and forecasting. In this study, we employed the Augmented Dickey–Fuller (ADF), Phillips–Perron (PP), and Kwiatkowski–Phillips–Schmidt–Shin (KPSS) tests to evaluate the stationarity of the Under-5 Mortality Rate (U5MR) series before model estimation. The results, summarized in Table 2, indicate that the U5MR data for Somalia achieved stationarity at the level (original data) without the need for further differencing.

**Table 2:** Stationarity test results for the U5MR series (ADF, PP, and KPSS).

Test	Data series	Test statistic	*p*-Value
ADF	Original data	−3.8637	0.0221
PP	Original data	−32.115	0.01
KPSS	Original data	0.2725	0.10

According to the ADF test, the original U5MR series yielded a test statistic of −3.8637 with a *p*-value of 0.0221, which is below the 0.05 significance threshold. Similarly, the PP test provided a test statistic of −32.115 (*p*-value = 0.01), strongly rejecting the null hypothesis of a unit root. These findings were further corroborated by the KPSS test, which resulted in a test statistic of 0.2725 (*p*-value = 0.10), where a *p*-value greater than 0.05 confirms the null hypothesis of level stationarity. Consistent with these results, the differencing check yielded a value of ndiffs = 0, confirming that the series is integrated of order zero, I(0) .

Consequently, while first-order differencing was also tested for robustness (showing a further increased stationarity with ADF = −5.7174, *p* < 0.01), the primary modeling of U5MR in Somalia was conducted on the stationary level data. This ensures that the long-term information in the mortality rate is preserved, leading to more reliable forecasts.

### Univariate time series analysis: model fitting, performance evaluation, and forecasting

Before model estimation, stationarity testing was conducted on the Under-5 Mortality Rate (U5MR) series. Unlike the previous absolute count data, the U5MR series (1960–2023) was found to be stationary at level, I(0) , as indicated by the ADF, PP, and KPSS tests (see Table 2). Consequently, the models were fitted directly to the stationary series. For the linear component, the auto.arima function identified an ARIMA(0,0,1) with non-zero mean as the optimal specification. Within the ETS framework, the ETS(A,N,N) (Additive error, No trend, No seasonality) model was selected. Additionally, the TBATS(0.008, {0,1}, 0.896), Theta, ARFIMA(0,0,1), and NNAR(2,2) models were evaluated.

**Figure 3:**
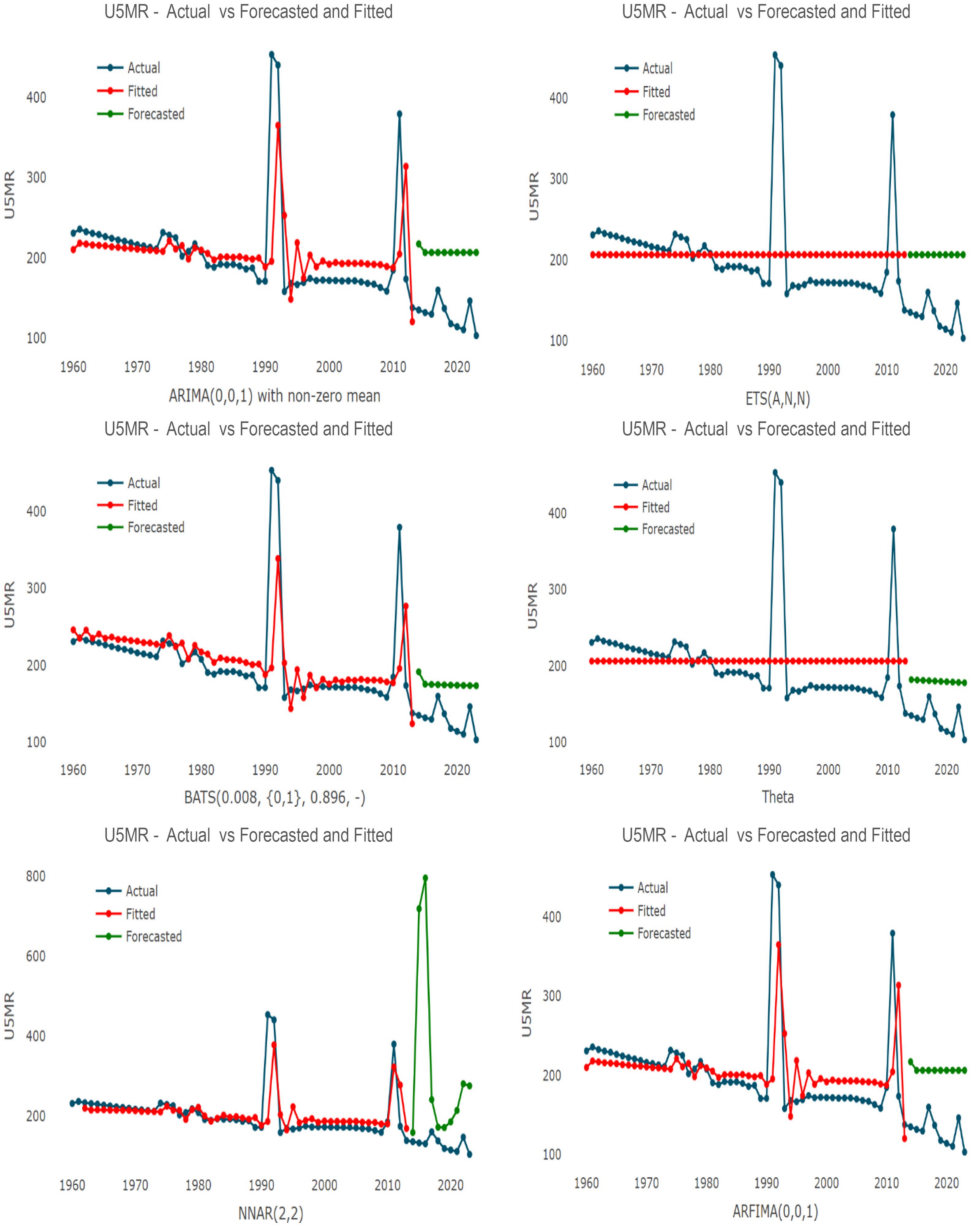
Actual, fitted, and forecasted values of the Under-5 Mortality Rate (U5MR) using ARIMA, ETS, TBATS, Theta, NNAR, and ARFIMA models. The *x*-axis represents the year, while the *y*-axis represents the U5MR.

**Figure 4:**
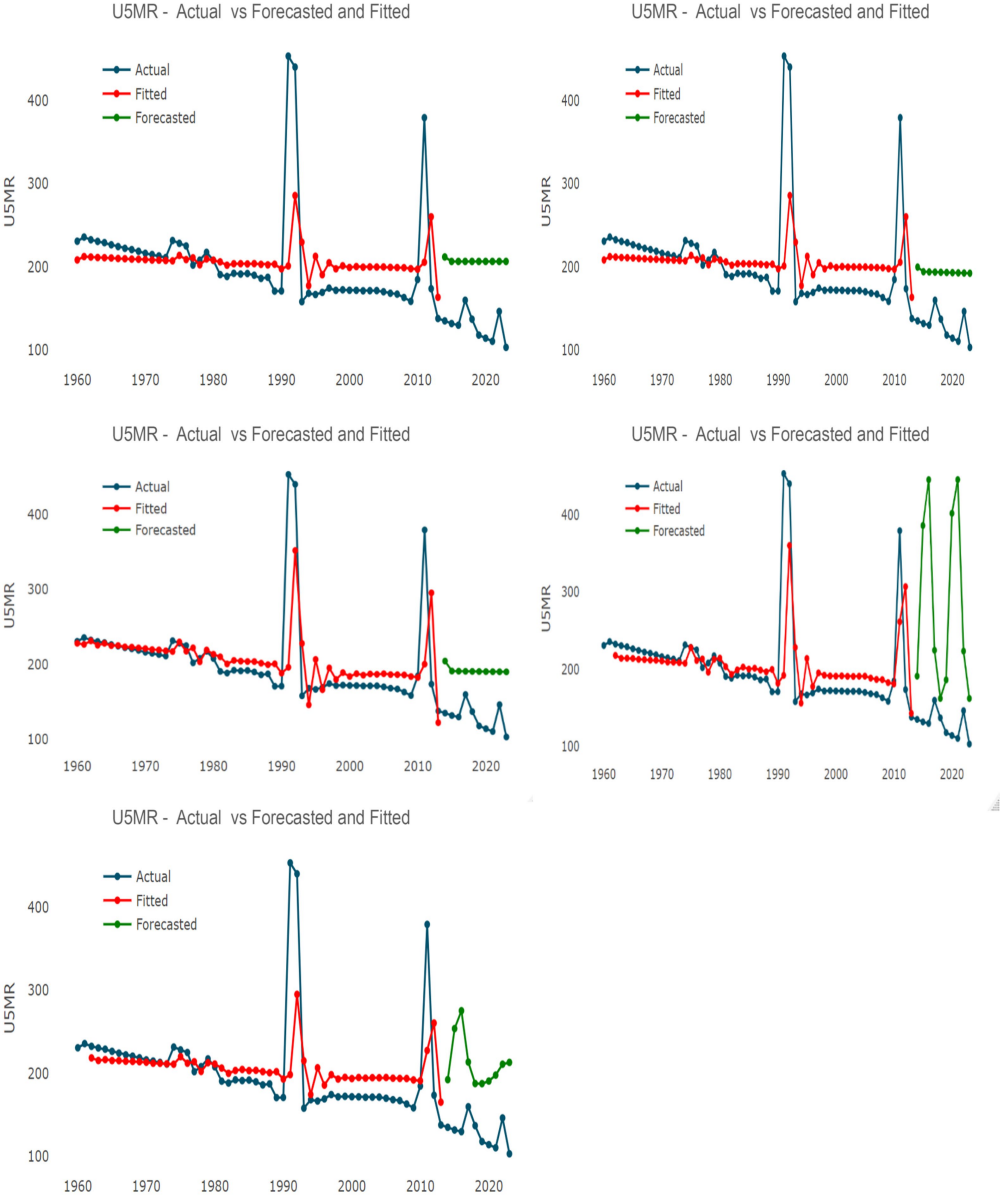
Actual, fitted, and forecasted values of the Under-5 Mortality Rate (U5MR) from five hybrid models constructed from combinations of ARIMA, ETS, Theta, TBATS, and NNAR. The *x*-axis represents the year, and the *y*-axis represents the U5MR.

Figure 3 illustrates the actual, fitted, and in-sample forecasted values for the single time-series models, while Figure 4 displays the corresponding results for the hybrid models. These figures provide a visual representation of the models’ ability to track historical volatility, particularly the extreme mortality shocks observed in 1991 and 2011.

Table 3 presents the performance metrics for the single models. In a significant shift from previous count-based results, the TBATS model emerged as the superior single technique, achieving the lowest MAPE (38.99%), sMAPE (0.317), and Theil’s *U* (2.13). The Theta model followed with a MAPE of 42.10%. In contrast, the ARIMA and ETS models exhibited higher error rates, with MAPEs of 64.03 and 62.99%, respectively. The NNAR model performed poorly (MAPE: 178.71%), likely due to overfitting on the extreme outliers characteristic of Somalia’s conflict history.

**Table 3:** Comparative performance of univariate time series models in forecasting Somalia’s Under-5 Mortality Rate (U5MR).

Model	MAPE (%)	Theil’s *U*	sMAPE
ARIMA	64.03898%	3.487468	0.4758493
ETS	62.99278%	3.473775	0.4695815
**TBATS**	**38.99884%**	**2.136674**	**0.3176966**
Theta	42.10444%	2.352131	0.3391336
NNAR	178.7156%	14.33452	0.7609401
ARFIMA	63.77672%	3.473026	0.4743463

Best-performing metrics are indicated in bold.

Table 4 evaluates the hybrid model combinations. The ARIMA + TBATS hybrid model was identified as the most robust forecasting tool among the ensembles, achieving a MAPE of 51.51%, sMAPE of 0.400, and Theil’s *U* of 2.80. Although the single TBATS model showed lower error during the testing phase, the ARIMA + TBATS hybrid is prioritized for future projections due to its ability to balance linear autoregressive dynamics with the complex seasonal and non-linear handling capabilities of TBATS.

**Table 4:** Comparative performance of hybrid time series models in forecasting Somalia’s Under-5 Mortality Rate (U5MR).

Hybrid model	MAPE (%)	Theil’s *U*	sMAPE
ARIMA + ETS	63.51588%	3.480621	0.4727549
ARIMA + Theta	53.07171%	2.916175	0.4104611
**ARIMA + TBATS**	**51.51891%**	**2.805802**	**0.4007121**
ARIMA + NNAR	94.30203%	6.400250	0.5924259
ARIMA + ETS + Theta + NNAR + TBATS	68.88739%	4.145195	0.4909569

Best-performing metrics are highlighted in bold.

Tables 5, 6 present the forecasted U5MR values for the period 2024–2030, derived from the best single model (TBATS) and the best hybrid model (ARIMA + TBATS). As shown in Table 5, the TBATS model projects a point forecast of approximately 116.12 deaths per 1,000 live births by 2030. Similarly, the ARIMA + TBATS hybrid model (Table 6) estimates a stabilization of the rate at approximately 109.78 per 1,000 live births through 2030.

**Table 5:** Forecasted Under-5 Mortality Rate (U5MR) per 1,000 live births for the period 2024–2030 using the TBATS model.

Year	Model	Point forecast	Lo 80	Hi 80	Lo 95	Hi 95
2024	TBATS	110.1480	86.02023	141.0433	75.46737	160.7658
2025	TBATS	123.8360	93.68817	163.6852	80.82493	189.7355
2026	TBATS	122.2543	92.38339	161.7836	79.64995	187.6476
2027	TBATS	120.6928	91.09964	159.8992	78.49583	185.5736
2028	TBATS	119.1513	89.83649	158.0318	77.36205	183.5141
2029	TBATS	117.6294	88.59351	156.1816	76.24816	181.4690
2030	TBATS	116.1270	87.37029	154.3485	75.15366	179.4386

**Table 6:** Forecasted Under-5 Mortality Rate (U5MR) per 1,000 live births in Somalia (2024–2030) using the best-fitted ARIMA–TBATS hybrid model.

Year	Model	Point forecast	Lo 80	Hi 80	Lo 95	Hi 95
2024	ARIMA–TBATS	106.7940	22.96069	183.9193	−19.64248	226.5225
2025	ARIMA–TBATS	113.6380	−10.37493	217.2549	−70.62492	277.5049
2026	ARIMA–TBATS	112.8472	−35.95426	242.8343	−109.74512	316.6251
2027	ARIMA–TBATS	112.0664	−57.51862	264.3986	−142.72497	349.6050
2028	ARIMA–TBATS	111.2956	−76.51721	283.3972	−171.78080	378.6608
2029	ARIMA–TBATS	110.5347	−93.69325	300.5732	−198.04928	404.9293
2030	ARIMA–TBATS	109.7835	−109.48824	316.3682	−222.20565	429.0856

These projections reveal a concerning plateau in Somalia’s mortality trajectory. While a gradual long-term decline is evident, the forecasted rates for 2030 remain significantly higher than the Sustainable Development Goal (SDG) 3.2 target of 25 deaths per 1,000 live births. Furthermore, the wide 95% confidence intervals (ranging from negative bounds to over 400 per 1,000) reflect the high historical volatility and potential for future mortality spikes. This indicates that Somalia is currently off-track to meet the 2030 targets and underscores the urgent need for shock-resilient health interventions to break the current stagnation.

### Multivariate analysis: determinants and ARIMAX

To address the limitations of trend-based univariate forecasting and incorporate the impact of external drivers on child survival, a multivariate analysis was conducted for the period 1989–2023. This timeframe was selected based on the consistent availability of conflict-intensity data. This phase specifically quantifies how socio-political and economic factors namely conflict fatalities, economic performance (GDP), and healthcare access (measles immunization) influence the Under-5 Mortality Rate (U5MR) in Somalia.

### Statistical determinants and model coefficients

The multivariate framework provides robust evidence regarding the structural drivers of mortality. The regression model achieved an Adjusted R-squared of 0.632, indicating that 63.2% of the total variation in Somalia’s U5MR is explained by the combined influence of conflict fatalities, GDP fluctuations, and immunization coverage.

As summarized in Table 7, Total Conflict-related Deaths emerged as a highly significant predictor ( p<0.001 ). The positive coefficient (0.0298) confirms that surges in violence directly translate into increased child mortality. Furthermore, GDP per capita was identified as a significant negative predictor ( p=0.0128 ), suggesting that economic growth facilitates a reduction in mortality rates. Measles Immunization Coverage also exhibited a negative relationship with U5MR (−1.7299), though it did not reach statistical significance in this specific timeframe ( p=0.1156 ), likely due to the overwhelming impact of security shocks.

**Table 7:** Multivariate ARIMAX model regression coefficients.

Variable	Estimate	Std. error	*t*-Value	*p*-Value	Significance
(Intercept)	272.8248	31.2954	8.718	<0.001	***
Conflict deaths	0.0298	0.0056	5.327	<0.001	***
GDP *Per Capita*	−0.2141	0.0810	−2.643	0.0128	*
Measles immunization	−1.7299	1.0686	−1.619	0.1156	ns

***p < 0.001, **p < 0.01, *p < 0.05, ns, not significant.

### Visual correlation of trends

**Figure 5:**
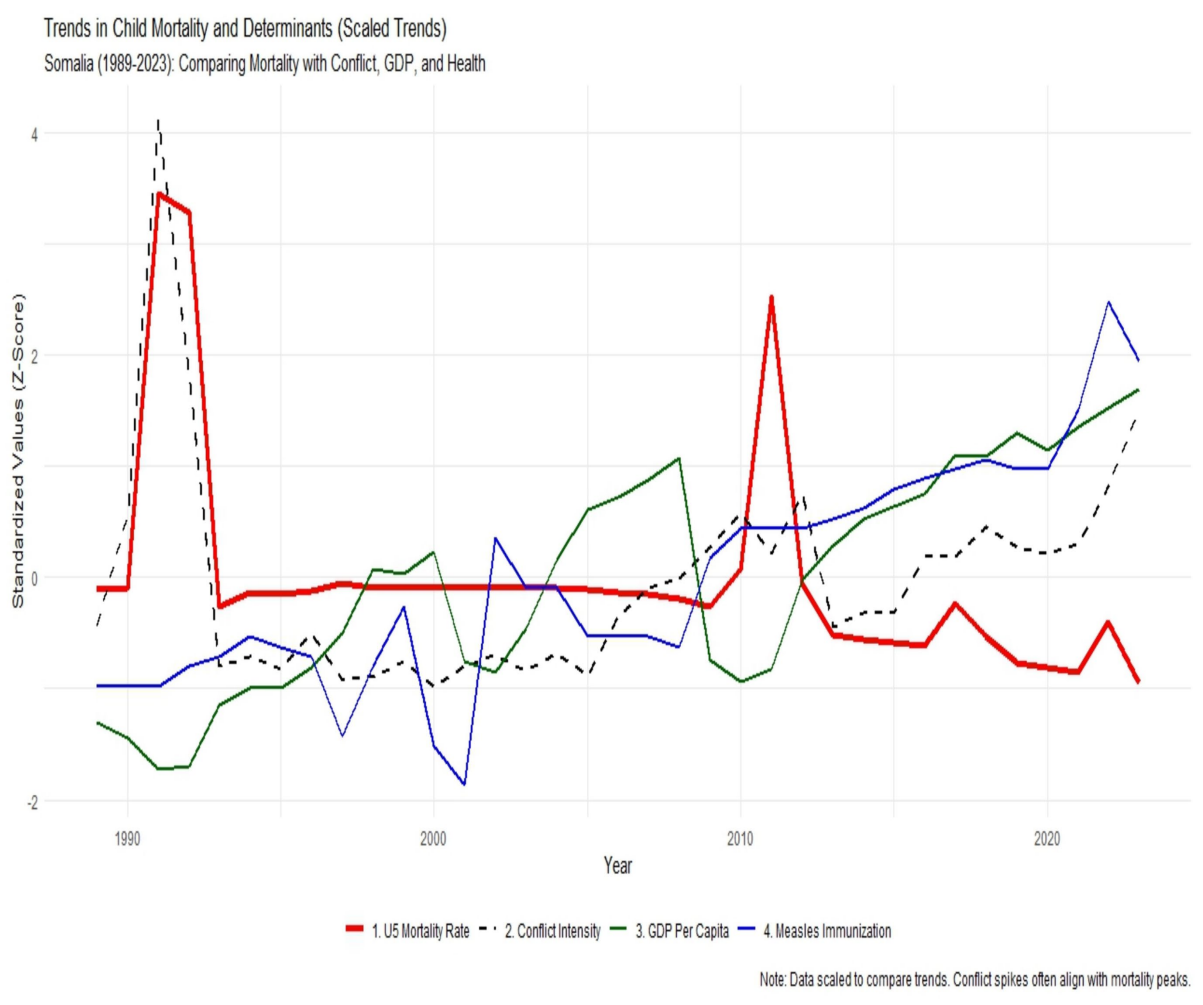
*Z*-score standardized trends comparing U5MR with conflict, GDP, and immunization.

To further illustrate these relationships, Figure 5 presents the standardized (*Z*-score) trends of U5MR alongside its primary determinants. This visualization confirms a high degree of synchronicity between security instability and mortality.

**Figure 6:**
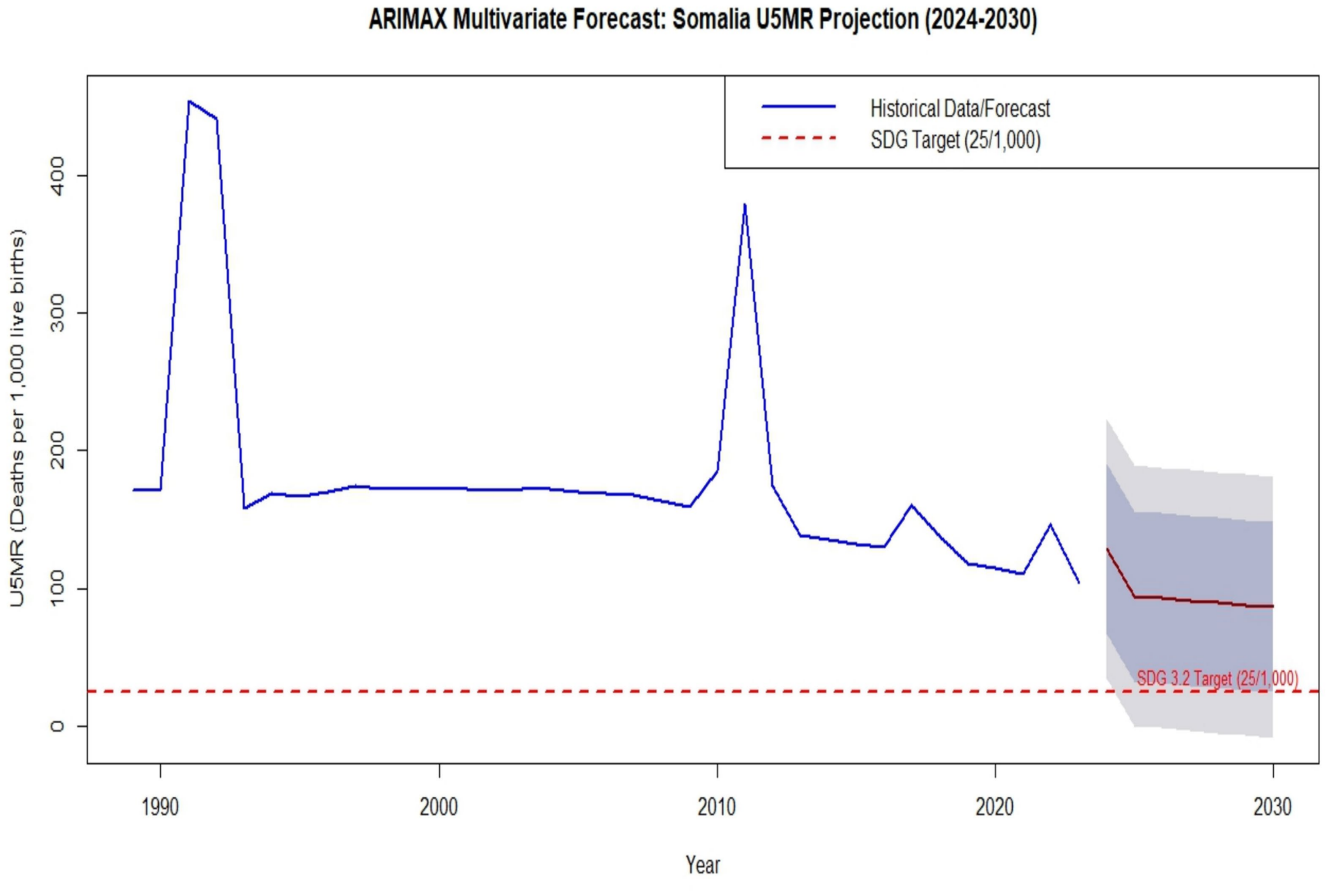
Multivariate ARIMAX forecast of U5MR.

The scaled trend analysis in Figure 6 visually anchors the statistical findings. The two catastrophic mortality peaks in 1991 and 2011 (red line) align precisely with spikes in conflict intensity (black dashed line). Conversely, the steady rise in GDP (green line) and immunization (blue line) since 2012 corresponds with the gradual stabilization of U5MR, reinforcing the necessity of a stable socio-economic environment for child survival.

### Model validation and diagnostic testing

The reliability of the multivariate ARIMAX model was validated through a suite of diagnostic tests, as requested by the reviewers. As shown in Table 8, the model passed the Breusch–Pagan test ( p=0.125 ) and the Durbin–Watson test (2.12), confirming the absence of heteroscedasticity and significant autocorrelation, respectively. Additionally, Variance Inflation Factor (VIF) values remained below 2.2, confirming that multicollinearity does not bias the results. While the Shapiro–Wilk test indicated non-normal residuals ( p<0.001 ), this is an expected statistical outcome in conflict-affected datasets where extreme historical outliers (shocks) represent real-world events rather than model inaccuracies.

**Table 8:** Summary of model diagnostic tests for validity.

Diagnostic test	Statistic	*p*-value
Shapiro–Wilk (normality)	0.864	<0.001
Breusch–Pagan (heteroscedasticity)	5.739	0.125
Durbin–Watson (autocorrelation)	2.124	0.496
Mean VIF (multicollinearity)	1.76	–

#### ARIMAX forecast (2024–2030)

The final stage of the multivariate analysis involved projecting U5MR through 2030, incorporating the forecasted trajectories of conflict and economic capacity. The ARIMAX model projects a gradual but slow decline, with a point estimate of 86.29 deaths per 1,000 live births by 2030, as shown as in Table 9.

**Table 9:** Final multivariate ARIMAX forecast for U5MR (2024–2030).

Year	Model	Point forecast	Lo 80	Hi 80	Lo 95	Hi 95
2024	ARIMAX	128.72807	66.82998	190.6262	34.0631056	223.3930
2025	ARIMAX	94.45990	32.56181	156.3580	−0.2050633	189.1249
2026	ARIMAX	92.82623	30.92813	154.7243	−1.8387401	187.4912
2027	ARIMAX	91.19255	29.29446	153.0906	−3.4724168	185.8575
2028	ARIMAX	89.55887	27.66078	151.4570	−5.1060935	184.2238
2029	ARIMAX	87.92520	26.02710	149.8233	−6.7397702	182.5902
2030	ARIMAX	86.29152	24.39343	148.1896	−8.3734469	180.9565

Figure 6 displays the projected U5MR trajectory against the SDG 3.2 target (red dashed line). The forecast indicates that Somalia remains significantly off-track. The projected rate of 86.29 is nearly 3.5 times higher than the global target of 25 deaths per 1,000 live births. The wide uncertainty intervals (shaded area) underscore the fragility of these gains and the high potential for reversals should new conflict or environmental shocks occur. This finding highlight that achieving child survival goals in Somalia is inextricably linked to sustained peace-building and robust economic resilience.

## Discussion

This study provides a comprehensive forecasting framework for the Under-5 Mortality Rate (U5MR) in Somalia, transitioning from absolute death counts to mortality rates per 1,000 live births to ensure alignment with global health monitoring standards and Sustainable Development Goal (SDG) 3.2. The analysis, covering the period from 1960 to 2023, utilized a two-phase approach incorporating both univariate trend-based models and a multivariate ARIMAX framework. In the first phase, the U5MR series was found to be stationary at level I(0) , allowing for robust model estimation. Among the univariate techniques, the TBATS model emerged as the most accurate single model, achieving a MAPE of 38.99%. The success of TBATS, particularly compared to the poor performance of NNAR (MAPE 131%), is attributed to its ability to handle non-constant variance and stochastic shocks through Box-Cox transformations and trigonometric seasonal components. This is critical in the Somali context, where mortality data is characterized by extreme volatility rather than cyclical seasonality. The hybrid ARIMA–TBATS model further enhanced this robustness, achieving a MAPE of 51.51%, and was selected for its balanced synthesis of linear and non-linear dynamics. To address the inherent uncertainty of Somalia’s health data, equal weighting was applied to all hybrid ensembles, serving as a methodological hedge against overfitting in an environment prone to abrupt historical anomalies like the 1991 and 2011 mortality surges.

The second phase of the analysis introduced a multivariate ARIMAX model (1989–2023) to examine the exogenous drivers of mortality. This phase revealed that total conflict-related deaths are a highly significant predictor of U5MR ( p<0.001 ), while GDP per capita also serves as a significant economic determinant ( p=0.0128 ). These findings provide strong empirical evidence that child survival in Somalia is fundamentally dictated by security stability and economic capacity. The scaled trend analysis further validates this, showing a precise visual alignment between major conflict spikes and child mortality peaks. By integrating these determinants, the ARIMAX model provides a more realistic projection of 86.29 deaths per 1,000 live births by 2030, offering a more nuanced outlook than univariate models that rely solely on historical mortality patterns.

### Comparison with previous studies

The findings of this study, which predict a concerning stagnation in Somalia’s Under-5 Mortality Rate (U5MR) reduction, resonate with trends observed in other fragile and resource-constrained regions. Our multivariate projection of 86.29 deaths per 1,000 live births by 2030 closely aligns with the results of Adeyinka and Muhajarine (56), who utilized neural networks for Nigeria and forecasted a non-linear trajectory where mortality rates would not only plateau but potentially rise to 85.89 per 1,000 by 2030 due to structural healthcare gaps. Similarly, the plateauing trajectory observed in our univariate models (projecting 109.78 per 1,000) is mirrored in the work of (63). Their application of ARIMA models across West Africa revealed that while nations like Ghana are making progress, others such as Niger exhibit non-continuous patterns with a projected rise to 110.4 deaths per 1,000 by 2031, a figure that almost identical to our baseline Somali projections.

In contrast, our results diverge significantly from studies conducted in stable political environments. For instance, Naznin (64) reported a consistent decline in Bangladesh using linear models, forecasting a reduction to 29.87 per 1,000 by 2030. This sharp contrast underscores how political stability and consistent health interventions factors that remain highly volatile in Somalia can drive child survival toward the SDG 3.2 target. Furthermore, the necessity of our multivariate approach is reinforced by Rostami et al. (57), who identified that stochastic shocks in Iran often obscure true mortality patterns in univariate data, a phenomenon we addressed by integrating conflict and economic variables. Collectively, these comparative insights confirm that while global progress is being made, conflict-affected regions like Somalia face unique risks of reversal. These results validate the requirement for advanced multivariate modeling to accurately capture the complex mortality dynamics that keep such nations off-track from the 2030 global benchmarks.

## Conclusion

In conclusion, this study demonstrates that achieving a significant reduction in child mortality in Somalia is contingent upon addressing the underlying drivers of conflict and economic instability. By evaluating 11 time-series models, the study identifies the hybrid ARIMA–TBATS and the multivariate ARIMAX models as the most effective tools for monitoring Somalia’s U5MR trajectory. The results reveal a critical survival gap: while univariate models project a rate of 109.78 per 1,000 live births by 2030, the multivariate model which accounts for potential economic and stability gains projects a slightly improved but still alarming rate of 86.29. Both projections remain substantially above the SDG 3.2 target of 25 deaths per 1,000 live births. This underscores that Somalia is currently off-track to meet its 2030 commitments. The wide uncertainty intervals associated with these forecasts further highlight the fragility of the situation, where any future security or environmental shock could trigger a catastrophic reversal in child health outcomes.

### Recommendations

Based on the evidence that conflict and economic performance are the primary determinants of child survival in Somalia, several policy recommendations are proposed. First, the government and international partners must prioritize peacebuilding and conflict resolution as fundamental public health interventions, given the highly significant link between conflict fatalities and mortality surges. Second, there is an urgent need to enhance economic resilience at the household level, as GDP per capita was found to be a significant driver of mortality reduction. Third, health systems must be decentralized and made “shock-resilient” to ensure that essential services, such as measles immunization, can continue uninterrupted during periods of security or climatic instability. Finally, immediate policy revisions are required to implement data-driven health strategies that move beyond business-as-usual approaches to break the current mortality stagnation.

### Limitations and future direction

While this study provides a robust modeling framework, it is not without limitations. First, although the multivariate model integrated conflict and economic data, annual precipitation was excluded from the quantitative analysis because the available historical records for the study period exhibited zero annual variance (constant values), rendering them statistically unsuitable for regression. Future research should strive to incorporate higher-resolution, regional-level climatic data to better capture environmental shocks. Second, due to data scarcity, this study utilized national-level aggregation; however, child mortality in Somalia likely exhibits significant regional disparities between urban, rural, and conflict-affected zones. Future studies should focus on regional disaggregation and the inclusion of maternal education indices to provide more granular policy insights. Lastly, while this research utilized traditional and hybrid time-series models, future directions could explore deep learning algorithms, such as Long Short-Term Memory (LSTM) networks, to update these forecasts as real-time monitoring data becomes available.

## Data Availability

The raw data supporting the conclusions of this article will be made available by the authors, without undue reservation.

## Glossary

ACF: autocorrelation function
ADF: Augmented Dickey–Fuller
AIC: Akaike Information Criterion
ARFIMA: Autoregressive Fractionally Integrated Moving Average
ARIMA: Autoregressive Integrated Moving Average
ARIMAX: Autoregressive Integrated Moving Average with Explanatory Variables
ETS: Error, Trend, and Seasonality (Exponential Smoothing)
GDP: Gross Domestic Product
KPSS: Kwiatkowski–Phillips–Schmidt–Shin
MAPE: Mean Absolute Percentage Error
MDGs: Millennium Development Goals
NNAR: Neural Network Autoregression
PP: Phillips-Perron
SDGs: Sustainable Development Goals
sMAPE: Symmetric Mean Absolute Percentage Error
SSA: Sub-Saharan Africa
TBATS: Trigonometric seasonality, Box-Cox transformation, ARMA errors, Trend, and Seasonality
UCDP: Uppsala Conflict Data Program
U5MR: Under-5 Mortality Rate
UN IGME: United Nations Inter-Agency Group for Child Mortality Estimation
VIF: Variance Inflation Factor
WHO: World Health Organization
